# Intake of Saturated Fatty Acids Affects Atherogenic Blood Properties in Young, Caucasian, Overweight Women Even without Influencing Blood Cholesterol

**DOI:** 10.3390/ijerph15112530

**Published:** 2018-11-12

**Authors:** Jadwiga Hamułka, Dominika Głąbska, Dominika Guzek, Agnieszka Białkowska, Agnieszka Sulich

**Affiliations:** 1 Department of Human Nutrition, Faculty of Human Nutrition and Consumer Sciences, Warsaw University of Life Sciences (SGGW-WULS), 159C Nowoursynowska Street, 02-787 Warsaw, Poland; agnieszka.bialkowska@szpitalczerniakowski.waw.pl (A.B.); agnieszka.sulich@gmail.com (A.S.); 2 Department of Dietetics, Faculty of Human Nutrition and Consumer Sciences, Warsaw University of Life Sciences (SGGW-WULS), 159C Nowoursynowska Street, 02-787 Warsaw, Poland; dominika_glabska@sggw.pl; 3 Department of Organization and Consumption Economics, Faculty of Human Nutrition and Consumer Sciences, Warsaw University of Life Sciences (SGGW-WULS), 159C Nowoursynowska Street, 02-787 Warsaw, Poland; dominika_guzek@sggw.pl; 4 Internal Department, Czerniakowski Hospital, 19/25 Stępińska Street, 00-739 Warsaw, Poland

**Keywords:** saturated fatty acids (SFA), monounsaturated fatty acids (MUFA), polyunsaturated fatty acids (PUFA), total cholesterol blood level, low-density lipoprotein (LDL), high-density lipoprotein (HDL), triglyceride, Atherogenic Index of Plasma (AIP), Caucasian women, overweight women

## Abstract

Despite a general relation between fat intake and cardiovascular risk factors, the association is often not observed in studies conducted in heterogenic populations, as for population groups, it may differ. The aim of the study was to analyze the associations between dietary fat intake and lipoprotein cholesterol fractions, as well as atherogenic blood properties, in young and middle-aged overweight Caucasian women. In a group of 138 overweight women, the three-day dietary records were assessed, under-reporters were excluded, and lipoprotein cholesterol fractions were analyzed. For the included 24 young (aged 20–40) and 42 middle-age women (aged 40–60), the intakes of fat, saturated fatty acids (SFA), monounsaturated fatty acids (MUFA), polyunsaturated fatty acids (PUFA), and cholesterol, as well as the PUFA/SFA ratio, were assessed. Afterwards, the analysis of associations with blood levels of total cholesterol (TC), low-density lipoprotein (LDL), high-density lipoprotein (HDL), and triglyceride, as well as the TC/HDL ratio, HDL/LDL, ratio and Atherogenic Index of Plasma (AIP), were conducted. It was stated that the influence of the dietary fat level on lipoprotein cholesterol fractions as well as atherogenic blood properties in overweight Caucasian women is age dependent. For young, overweight, Caucasian women, the influence of the dietary fat level on the lipoprotein cholesterol fractions was not observed; however, SFA intake influenced atherogenic blood properties. For middle-aged, overweight, Caucasian women, the PUFA intake had an especially important influence in increasing the HDL cholesterol level. For overweight Caucasian women, not only should lipoprotein cholesterol fractions be controlled, but also the AIP calculated—especially for younger women.

## 1. Introduction

The influence of dietary fat and cholesterol intake on cardiovascular risk has been analyzed for years [1]. That a general association exists is well known, and the American Heart Association (AHA) [2] recommends to reduce the intake of saturated fatty acids (SFAs) and, instead, to increase the intake of monounsaturated and polyunsaturated fatty acids (MUFAs and PUFAs, respectively). However, a number of studies indicate a lack of a statistically significant influence, and even systematic reviews and meta-analysis prove that the influence may not be strong enough to be statistically significant. In a meta-analysis of prospective epidemiologic studies by Siri-Tarino et al. [3], it was observed that the association between SFAs and increased risk of coronary heart disease or cardiovascular disease cannot be concluded and more data are needed. Similarly, in the systematic review by Clifton and Keogh [4], it was reported that intake of SFA is associated with neither coronary heart disease events nor cardiovascular disease mortality. Furthermore, in the systematic review and meta-analysis of observational studies by de Souza et al. [5], it was observed that intake of SFAs was not associated with coronary heart disease, cardiovascular disease, and all-cause mortality.

The indicated lack of a significant association is attributed to the fact that when reducing intake of fats or SFAs, patients simultaneously change their general dietary habits and these should be also monitored [4]. However, the lack of association is attributed primarily to the methodological limitations related to a lack of sufficient numbers of studies conducted [3] and, as a result, the heterogeneous groups analyzed and heterogeneous studies included in the analysis [5]. Taking this into account, it may be surmised that the effect of fat intake on cardiovascular risk may differ in various population groups, whereas specific groups at increased risk should be especially taken into account.

At the same time, a quantitative meta-analysis of metabolic ward studies by Clarke et al. [6] indicated that replacing 60% of SFAs by other fats and removing 60% of cholesterol from the diet reduces the blood cholesterol level by 10–15% and, especially, reduces low-density lipoprotein (LDL) cholesterol blood levels (80% of reduction). However, despite this conclusion, the other observations often did not prove the association in practice in specific groups of patients. In particular, in a group of overweight and obese adults, a recent systematic review and meta-analysis of randomized control trials by Hannon et al. [7] indicated that, in eight of the studies that were included and conducted in a group of 663 patients, there was no significant influence of the replacement of SFAs by unsaturated fatty acids on the correction of the blood lipid profile.

Based on the indicated results, one could decide about the necessity to abandon recommending changes of dietary fat intake, as there is a little evidence that it may benefit lipid profiles in this population and there is no evidence that it may reduce cardiovascular risk. However, as previously indicated, the heterogeneity of studies may influence the observed results [5]. In the systematic review and meta-analysis by Hannon et al. [7], it was concluded that studies should be conducted for groups of various ages, ethnicities, or genders; the heterogeneous groups analyzed so far may be considered the reason for inconclusive results. Moreover, obese individuals, while compared with overweight ones, may be characterized by more serious cardiovascular disturbances, as subclinical vascular disease is commonly reported [8], so combining overweight and obese ones may also contribute to obtaining heterogeneous groups.

The aim of the study was to analyze the associations between dietary fat intake and lipoprotein cholesterol fractions, as well as atherogenic blood properties, in young and middle-aged overweight Caucasian women. The objective was to analyze the influence of intakes of fat, SFA, MUFA, PUFA, and cholesterol, as well as the PUFA/SFA ratio, on the blood levels of total cholesterol (TC), low-density lipoprotein (LDL), high-density lipoprotein (HDL), and triglyceride, as well as the TC/HDL ratio, HDL/LDL ratio, and Atherogenic Index of Plasma (AIP).

## 2. Materials and Methods

### 2.1. Ethical Commission Agreement

The study was approved by the Bioethical Commission of the National Food and Nutrition Institute (No 1805/2011). The written constant agreement was obtained from all participants and the study was conducted in compliance with the Declaration of Helsinki.

### 2.2. Studied Group

The recruitment procedure was conducted in the Outpatient Clinic of the Czerniakowski Hospital in Warsaw. Patients of the general outpatient clinics were informed about the planned study as well as the inclusion and exclusion criteria. Moreover, they were informed that each participant would receive her blood test results, as well as information about the nutritional value of their diet.

This study was planned to be conducted in a group of overweight women (body mass index—BMI Є <25.0–30.0 kg/m^2^>), as it was to be analyzed for excessive body mass individuals—an increased-cardiovascular-risk group [9]. However, obese individuals were not planned for inclusion as they are commonly characterized by more serious disturbances, not merely a risk and, moreover, subclinical vascular disease is commonly reported [8]. 

The following inclusion criteria were specified:

- Caucasian women;
- age: 20–60 years;
- menstruating;
- overweight;
- three-day dietary record prepared; and
- informed written consent to participate.

The following exclusion criteria were defined:

- Pregnancy or lactation;
- diet-related diseases diagnosed;
- allergies and food intolerance;
- hypolipemic drugs;
- women undergoing body mass loss; and
- women on any special diet.

The list of allowed diseases and medical conditions (other than diet-related ones that were the reason to exclude participants) was prepared and among the allowed ones, according to the ICD-10 [10], there were the following categories: A00-B99 (infectious and parasitic diseases), F00-F99 (mental and behavioral disorders), if not impairing the cognitive function, G00-G99 (diseases of the nervous system), H00-H59 (diseases of the eye and adnexa), H60-H59 (diseases of the ear and mastoid process), J00-J99 (diseases of the respiratory system), L00-L99 (diseases of the skin and subcutaneous system), M00-M99 (diseases of the musculoskeletal system and connective tissue), and N00-N99 (diseases of the genitourinary system). To not cause exclusion, the diagnosed diseases were to be indicated as chronic and not diet-related.

Because reliable assessment of diet was the basis of the planned analysis, all women considered to under-report their dietary intake were also excluded on the basis of their three-day dietary record. For each respondent, the energy value of the declared typical diet was calculated and it was compared with the individual basal metabolic rate, calculated using the Harris–Benedict equation [11]. If lower, the overweight individual was treated as an under-reporter and excluded from further analysis.

**Figure 1:**
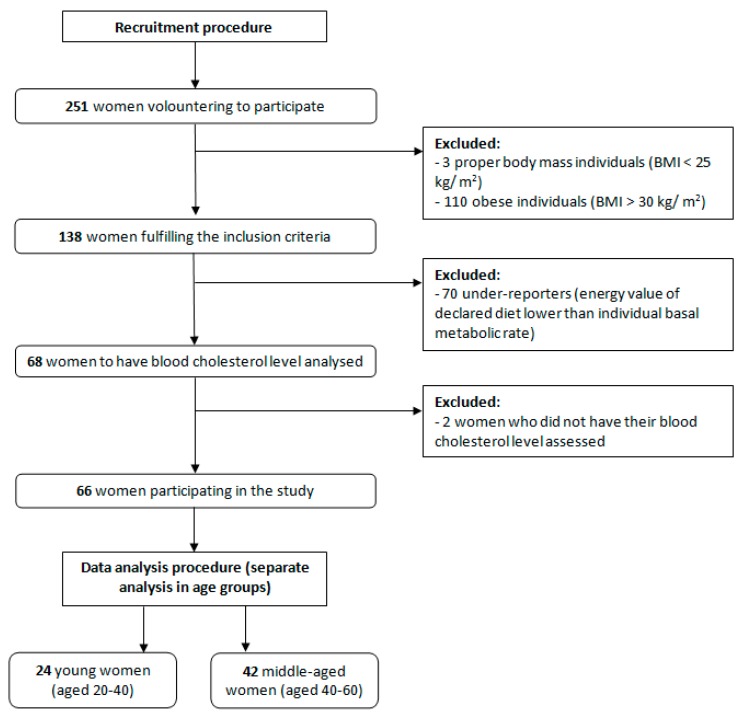
Flow chart of the inclusion procedure and study design.

The inclusion procedure and study design are presented in Figure 1. After including participants into the study, two separate age groups were formulated—women younger than 40 (*n* = 24) and older than 40 (*n* = 42)—to compare associations observed in two groups and observe potential age-dependent relations. However, all procedures conducted in both groups were identical, and they were treated separately only in analysis. The age of 40 was chosen in order to divide the study sample into younger and older ones, as in a number of studies of cardiovascular risk, the participants aged above 40 are treated as a separate group, as they may develop cardiovascular problems even if not diagnosed [12,13]. Similarly, for the Framingham Heart Study cohort, the age group of participants older than 40 was also analyzed [14] and some authors even recommend to conduct the formal cardiovascular disease risk assessment, in the case of no risk factors, only for patients at the age of 40 or more [15].

The frequency of chronic not diet-related diseases was compared between groups of included participants, as well as the general characteristics of groups was also compared and stated to be not differing (Table 1).

### 2.3. Body Mass Assessment

The anthropometric measurements were undertaken by a registered dietitian, according to the international standards for anthropometric assessment guidelines [16]. Height and body weight were measured to the nearest 0.1 cm and 0.1 kg, respectively, with the participants in lightweight clothing and without shoes.

The BMI was calculated and categorized according to the World Health Organization (WHO) stratification [17]. Participants characterized by BMI Є <25.0–30.0 kg/m^2^> were interpreted as overweight.

The anthropometric parameters were compared between groups of included participants and stated to be not differing (Table 2).

### 2.4. Diet Assessment

The diet was assessed by a registered dietitian, based on a three-day dietary record that was conducted by respondents before the study qualification (while volunteering to participate, candidates were asked to come to the qualifying researcher with their dietary record). They received all necessary information about the principles of conducting a dietary record and were informed about the necessity to scrupulously record all food products consumed and beverages drunk during the specified period. Furthermore, they received a structured format together with the necessary instructions to record all consumed meals with their time and location of consumption, all ingredients, and the serving size. The records were to be maintained during three typical, random, and not successive days; two of them were to be weekdays and one was to be a weekend day. When recording the serving size, respondents were asked to use a kitchen scale and note the weight of the serving (if they had kitchen scales), or to estimate the serving as typical household measures (if they did not have kitchen scales). All the servings were verified by a researcher during qualification and, once again, by a dietitian, using the Polish food model booklet [18].

The intake of food products was calculated as a mean from the three recorded days to obtain a mean daily intake for each participant, while the following food product groups were considered: Dairy beverages, low-fat dairy products (e.g., cottage cheese, curd), high-fat dairy products (e.g., hard cheese, processed cheese), meat, meat products (e.g., cold cuts, smoked meat), fish and fish products, egg, plant fats (e.g., oil, margarine), and dairy fats (e.g., butter, cream). The additional analysis of the between-group comparison of the food products’ intake in a diet of participants is presented in the supplementary material (Table S1). Similarly, in the supplementary material, there is an analysis of the correlation between food products’ intake and blood levels of TC (Table S2), LDL (Table S3), HDL (Table S4), TC/HDL ratio (Table S5), HDL/LDL ratio (Table S6), triglyceride (Table S7), and AIP (Table S8), stratified by age.

The intake of nutrients was calculated as a mean from the three recorded days to obtain a mean daily intake for each participant, after considering technological losses, by using the nutritional software, *Dieta* (version 5.0.) [19], which is based on Polish food-composition tables [20]. The following nutrients were calculated and included for further analysis: Fat intake (g), fat share (% of energy value of diet), SFA intake (g), SFA share (% of energy value of diet), MUFA intake (g), MUFA share (% of energy value of diet), PUFA intake (g), PUFA share (% of energy value of diet), n-3 PUFAs: alpha-linolenic acid (ALA—18:3) (g), eicosapentaenoic acid (EPA—20:5) (g), docosahexaenoic (DHA—22:6) (g), total n-6 PUFA (calculated as a sum of linoleic acid (LA—18:2), gamma-linolenic acid (GLA—18:3), arachidonic acid (ARA—20:4)) (g), cholesterol intake (mg), and the PUFA/SFA ratio. Due to the fact, that trans fatty acids content is not presented in Polish food-composition tables [20], to calculate it and include it for further analysis, it was necessary to use the United States Department of Agriculture food-composition tables (version 3.9.5.1.) [21]. 

In addition, the energy value of the diet was used to assess the credibility of respondents and to enable the exclusion of under-reporters, and the protein and carbohydrates intake were also calculated to enable presentation of the characteristics of the diets.

The general characteristics of diets were compared between groups of included participants and stated to be not differing (Table 3).

### 2.5. Blood Lipid Profile Assessment

Venous blood samples were collected after overnight fasting (12 h) in the morning (9–10 a.m.) with minimal stasis, and then maintained at 4 °C until plasma or serum was separated for biochemical analyses. Plasma and serum samples were collected after centrifugation (1000× *g* for 10 min at 4 °C) and stored frozen (−80 °C) until analysis for the assessment of lipoproteins’ composition and concentration in blood plasma.

TC, HDL cholesterol, and triglyceride blood levels were determined through standard enzymatic analyses using commercial HYDREX kits (product numbers: Total cholesterol—HXB104; HDL cholesterol—HXB106; triglycerides—17628). The LDL cholesterol blood level was calculated using Friedewald et al.’s formula [22].

Based on the obtained results of TC, LDL cholesterol, HDL cholesterol, and triglyceride blood levels, the following parameters were calculated: TC/HDL ratio, HDL/LDL ratio, and AIP. The TC/HDL ratio and HDL/LDL ratio were calculated as a quotient of respective blood cholesterol fractions. The AIP was calculated as a logarithmically transformed ratio of molar concentrations of triglycerides to HDL blood levels [23].

After the blood lipid profile was analyzed, the values obtained were compared to the recommended reference blood lipid levels. The European guidelines on cardiovascular disease prevention in clinical practice [24] were applied as follows: TC < 190 mg/dL, LDL cholesterol < 115 mg/dL, HDL cholesterol > 45 mg/dL, and triglyceride < 150 mg/dL. For the TC/HDL ratio, as well as the HDL/LDL ratio, the commonly recommended values were accepted as follows: TC/HDL ratio < 4.0 [25], and HDL/LDL ratio > 0.3 [26]. For AIP, values < 0.11 were treated as the recommended values, whereas values 0.11–0.21 and >0.21 were treated as being associated with a moderate and high cardiovascular risk, respectively [27].

### 2.6. Statistical Analysis

To assess the normality of distributions, the Shapiro–Wilk test was applied. Between-group comparisons were undertaken with the Student’s *t*-test (for parametric distributions) and Mann–Whitney *U* test (for nonparametric distributions). To compare the share of individuals, the chi^2^ test was applied. To analyze the correlations between dietary fat intake and lipoprotein cholesterol fractions, as well as atherogenic blood properties, the Pearson’s correlation coefficient (for parametric distributions) and the Spearman’s rank correlation coefficient (for nonparametric distributions) were applied.

Statistical analysis was conducted using the Statistica software version 8.0 (StatSoft Inc., Tulsa, OK, USA), and *p* ≤ 0.05 was interpreted as a significant correlation.

## 3. Results

In the analyzed group of overweight women, no differences of dietary fat intake were observed between women from various age groups (*p* > 0.05; Student’s *t*-test, Mann–Whitney *U* test). Moreover, neither were differences of lipoprotein cholesterol fractions and atherogenic blood properties detected between age groups (*p* > 0.05; Student’s *t*-test, Mann–Whitney *U* test), nor were differences in the percentage of individuals characterized by the recommended level of lipoprotein cholesterol fractions observed between groups (Table 4).

The analysis of the correlation between TC blood level and dietary fat intake in overweight women, stratified by age, is presented in Table 5. No association between factors associated with dietary fat level and TC blood level was observed for overweight women, independent of the age group.

The analysis of the correlation between LDL cholesterol blood levels and dietary fat intake in overweight women, stratified by age, is presented in Table 6. No association between factors associated with dietary fat and LDL cholesterol blood levels was reported for overweight women, independently of the age group.

The analysis of the correlation between HDL cholesterol blood level and dietary fat intake in overweight women, stratified by age, is presented in Table 7. No association between factors associated with dietary fat and HDL cholesterol blood level was reported for the younger group of overweight women. At the same time, for middle-aged overweight women, the positive correlation between PUFA intake (% of energy value of diet) and HDL cholesterol blood level was statistically significant (*p* = 0.049; *R* = 0.3056).

The analysis of the correlation between TC/HDL blood cholesterol ratio and dietary fat intake in overweight women, stratified by age, is presented in Table 8. No association between factors associated with dietary fat level and TC/HDL blood cholesterol ratio was identified for the younger group of overweight women. However, for middle-aged overweight women, a negative correlation between the dietary PUFA/SFA ratio and TC/HDL blood cholesterol ratio was found to be statistically significant (*p* = 0.017; *R* = −0.3680).

The analysis of the correlation between the HDL/LDL blood cholesterol ratio and dietary fat intake in overweight women, stratified by age, is presented in Table 9. No association between factors associated with dietary fat level and HDL/LDL blood cholesterol ratio was observed for the younger group of overweight women. However, for middle-aged overweight women, a positive correlation between the dietary PUFA/SFA ratio and HDL/LDL blood cholesterol ratio was found to be statistically significant (*p* = 0.049; *R* = 0.3062).

The analysis of a correlation between triglyceride blood level and dietary fat intake in overweight women, stratified by age, is presented in Table 10. No association between factors associated with dietary fat level and triglyceride blood level was observed for overweight women, independently of the age group.

The analysis of the correlation between AIP and dietary fat intake in overweight women, stratified by age, is presented in Table 11. In a group of young overweight women, a positive correlation between SFA intake (g) (*p* = 0.040; *R* = 0.4223) and SFA intake as a percentage of the energy value of the diet (*p* = 0.031; *R* = 0.4417) with AIP was observed; moreover, a negative correlation between the dietary PUFA/SFA ratio and AIP (*p* = 0.019; *R* = −0.4757) was found to be statistically significant. However, in a group of middle-aged overweight women, a negative correlation between the dietary PUFA/SFA ratio and AIP (*p* = 0.011; *R* = −0.3901) was found to be statistically significant.

## 4. Discussion

A number of studies so far have indicated the role of blood cholesterol level monitoring in the prevention of cardiovascular diseases. Among others, it was proven in a meta-analysis of individual participant data by Paige et al. [28] that the repeated measurements of blood cholesterol level and blood pressure are significantly associated with cardiovascular risk prediction. The meta-analysis by Robinson et al. [29] indicated that non-HDL cholesterol blood level must be an important target in the treatment and prevention of coronary heart disease. Furthermore, a systematic review and meta-analysis by Peters et al. [30] indicated that total blood cholesterol level is a strong risk factor for coronary heart disease.

However, not only the blood lipid profile, but also other factors may influence cardiovascular risk. Besides, there is increasing evidence that body mass is a strong independent factor. Despite BMI being indicated as a factor significantly associated with all-cause mortality, which was confirmed from a meta-analysis of individual participants’ data comprising 239 prospective studies in four continents [31], a specific association with cardiovascular incidents and related mortality is emphasized. A general association between body mass and cardiovascular events has been reported [32], whereas BMI assessment combined with waist circumference measurement is indicated to have significant clinical benefits in cardiovascular risk assessment [33]. This results from the role of excessive body mass combined with abdominal adiposity in the increasing risk of heart failure, which was proven in a systematic review and dose–response meta-analysis of prospective studies by Aune et al. [34].

Taking it into account, in overweight and obese individuals, cardiovascular events’ prevention should include not only blood cholesterol monitoring, but also body mass reduction. It is accordingly recommended by the AHA, as indicated for the prevention of cardiovascular diseases, that a BMI lower than 25 kg/m^2^ should be maintained [2]. Moreover, it must be emphasized that the analyzed overweight patients may be treated as a group characterized by increased cardiovascular risk, independently from their blood cholesterol level. Furthermore, although only lipoprotein cholesterol fractions level parameters were analyzed, various cardiovascular risks were indicated depending on the parameter that was applied—the risk was highest for total cholesterol, LDL cholesterol, and AIP levels (atherogenic potential for 60–90% of respondents in both groups), whereas the lowest risk was noted for HDL cholesterol and the HDL/LDL ratio (atherogenic potential for 5–20% of respondents in both groups).

Due to the cardiovascular risk stated in the presented group, action necessary to decrease the elevated risk should be indicated in such patients. However, when the dietary factors were considered, various roles of diet were observed in the age-dependent subgroups of overweight women. Because the association between fatty acid intake and blood lipid profile was indicated in the meta-analysis of randomized control trials by Hannon et al. [7] as not significant in a group of excessive body mass individuals, and on the basis of the presented study conducted in a homogenous group, it may be hypothesized that such an association is significant, but only in older, and not younger, female respondents. In our study, it was observed that for middle-age overweight Caucasian female individuals, the PUFA share in the energy value of diet was positively correlated with HDL cholesterol blood level, whereas the PUFA/SFA ratio was negatively correlated with the TC/HDL ratio and positively with the HDL/LDL ratio—both proving the positive effect of increasing the proportion of PUFA in the total fat intake. However, the correlations denoted for middle-age respondents were not observed for younger respondents and were not even close to statistical significance.

The beneficial effect of PUFA fatty acids is well known, especially when their influence on the HDL cholesterol blood level is stated [35]. Moreover, the effect of PUFA increasing the HDL level is stated independently from the lipid profile [36]. It was observed in the study of Sanders and Hochland [37] that a supplement of a fish oil concentrate causes an increase of the HDL cholesterol levels in healthy individuals. Similarly, in a study of Simons et al. [38], the same observations were indicated for patients with hyperlipidaemia.

It is indicated that PUFA fatty acids have an impact on the reverse cholesterol transport mechanism, by which excessive cholesterol is transported from peripheral tissues to the liver for hepatobiliary excretion; this inhibits foam cell formation and reduces the development of atherosclerosis [39]. Furthermore, in the meta-analysis of control trials by Mensink et al. [40], an identical influence was observed to that in the present study because a replacement of other fatty acids by cis-PUFA had the most beneficial effect as it caused the largest decrease of the TC/HDL cholesterol ratio.

Moreover, a specific influence of n-3 PUFA supplementation on HDL cholesterol levels is in general observed [41], but it is may be secondary to a triglyceride level decrease [42] that is also commonly stated to be caused by n-3 PUFA intake [43]. The indicated effect of increasing HDL level is especially strong for DHA, but not for ALA or EPA [44]. Similarly, the DHA, but not EPA, is observed to increase HDL and LDL particle sizes [45], but the clinical significance of this fact is stated to be unclear [42]. However, in the presented study, such an effect of specific n-3 fatty acids was observed for none of the analyzed n-3 fatty acids, ALA, EPA, and DHA, and the influence was also not stated for the triglyceride blood level, only for total PUFA.

Nonetheless, the question arises as to why the indicated influence was not observed in the case of younger respondents and, in the present study, only for women older than 40 years was a beneficial effect of PUFA observed. It may be associated with the general decrease of PUFA blood level observed as age progresses, as was observed on the basis of observations from 160,000 patients for red blood cell membrane fatty acids [46]. In the study of Harris et al. [46], the influence of age was especially significant for LA for which, on the basis of the systematic review and meta-analysis of prospective cohort studies by Farvid et al. [47], an especially important dose–response inverse association with coronary heart disease risk was confirmed. Thus, the significant influence of PUFA intake on blood cholesterol in older respondents may be attributed to a natural decrease of blood PUFA levels and a resulting higher susceptibility to the influence of dietary PUFA. As a result, with increasing age, PUFA may become more important to counteract this natural decrease and regulate the levels of lipoprotein cholesterol fractions.

The other important observation from the present study is the lack of influence of dietary fat levels on lipoprotein cholesterol fractions in younger respondents, accompanied by the influence on the calculated AIP. Because the AIP is a strong marker for predicting the risk of atherosclerosis and coronary heart disease [48], and given its correlation with BMI [49], its role is also important. Moreover, it is calculated as a comprehensive factor, including both triglyceride and HDL cholesterol levels; therefore, it may be considered to be a better predictor than a single lipid parameter than is, in general, indicated for comprehensive lipid indexes [50,51] and specifically for AIP [52,53].

The presented study, conducted in a population of young, Caucasian, overweight women, showed that the dietary SFA intake and SFA share in the energy value of diet were positively correlated with AIP, whereas the PUFA/SFA ratio was negatively correlated with it, indicating the potential atherogenic influence of SFA intake and a beneficial influence of PUFA that was not observed when only single lipid parameters were analyzed. In the middle-aged population of Caucasian overweight women, an identical effect of the PUFA/SFA ratio was reported, confirming the previously indicated important influence of PUFA.

However, the results observed for AIP are especially important for the younger population. Although the influence was not observable when single lipid parameters were assessed, the influence of dietary fatty acid intake and its atherogenic potential cannot be denied. Therefore, it could be recommended, especially for younger women populations, to not only assess single lipid parameters, but to also calculate the AIP to analyze the potential influence of diet, which may not be observed with single parameters alone due to the potential influence of a natural higher PUFA blood level [46]. Such an approach may be an important aim for public health to obtain better cardiovascular risk prevention. Moreover, as for young women with no additional risk factors, the formal cardiovascular disease risk assessment may not be conducted [15], the control of such an additional parameter may be an easy way to monitor the risk in the most efficient way. Such an approach is in agreement with the statement that tailored health education for specific communities is needed to reduce coronary heart disease risk [54].

Despite important observations not indicated so far in other publications, the limitations of this study must be mentioned. The main limitation is the sample size; although the aim of the study was to assess diet in a homogenic group (Caucasian women; age groups: 20–40 and 40–60; BMI: 25–30 kg/m^2^), the sample size was supposed to be smaller than in a more heterogeneous group of respondents. Moreover, excluding all women considered as under-reporters was the other factor that significantly reduced the sample size, as every second participant was excluded; this is typical because under-reporting is commonly noted for excessive body mass participants [55]. At the same time, excluding all respondents considered as under-reporters must be confirmed as an important strength of the study, as the group was not only homogeneous, but also their dietary observations must be considered reliable. Moreover, the important purpose of the study was to conduct it not only in a homogenous group, but also for women (as majority of similar studies were so far conducted for the population of male respondents) and overweight individuals (as majority of conducted so far ones were performed for obese patients). Further studies should include larger groups of respondents, but the essential aim should be conducting analysis for homogenous groups.

## 5. Conclusions

1. The influence of dietary fat intake on lipoprotein cholesterol fractions as well as atherogenic blood properties in overweight Caucasian women is age dependent.
2. For young, overweight, Caucasian women, the influence of the dietary fat level on the lipoprotein cholesterol fractions may not be observed; however, SFA intake influences atherogenic blood properties.
3. For middle-aged, overweight, Caucasian women, the PUFA intake may have an especially important influence, as it causes an increase of the HDL cholesterol level.
4. For overweight Caucasian women, not only should lipoprotein cholesterol fractions be controlled, but also the AIP calculated—especially for younger women.

## Figures and Tables

**Table 1:** Between-group comparison of the general characteristics of participants (chi^2^ test).

Parameters		Number (%)		*p*
		Age < 40 Years	Age ≥ 40 Years	
Place of residence	Town below 100,000 inhabitants	5 (20.8)	5 (11.9)	0.6143
	Town above 100,000 inhabitants	19 (79.2)	27 (88.1)	
Chronic diseases	At least 1 disease diagnosed	10 (41.7)	20 (47.6)	0.6406
	Not diagnosed	14 (58.3)	22 (52.4)	
Vitamin/mineral supplementation	Yes	7 (29.2)	20 (47.6)	0.1425
	No	17 (70.8)	22 (52.4)	

**Table 2:** Between-group comparison of the anthropometric parameters of participants (Student’s *t*-test).

Parameters	Age < 40 Years		Age ≥ 40 Years		*p*
	Mean ± SD	Median (Range)	Mean ± SD	Median (Range)	
Age (years)	30.4 ± 6.4	29.5 * (20.0–39.0)	52.1 ± 5.6	54.0 * (41.0–60.0)	Not applicable
BMI (kg/m^2^)	27.6 ± 1.2	27.5 (25.3–29.8)	27.4 ± 1.4	27.6 (25.2–29.8)	0.4620

* the distribution different than normal (verified using Shapiro-Wilk test—*p* ≤ 0.05).

**Table 3:** Between-group comparison of the general characteristics of the diets of participants.

Parameters	Age < 40 Years		Age ≥ 40 Years		*p* **
	Mean ± SD	Median (Range)	Mean ± SD	Median (Range)	
Energy (kcal)	2195.0 ± 311.3	2126.1 * (1824.6–3021.9)	2123.8 ± 309.8	2070.2 * (1581.4–3034.7)	1.0000
Energy (kcal/kg of BM)	28.9 ± 5.2	28.5 (20.4–40.4)	29.8 ± 5.0	28.2 * (23.4–46.3)	0.5179
Fat (g)	74.8 ± 19.4	68.1 * (56.3–128.3)	74.3 ± 20.7	72.3 * (38.4–140.4)	0.7239
Fat (% of energy value)	30.6 ± 6.2	29.7 * (17.8–52.7)	31.4 ± 6.8	30.7 (18.5–45.5)	0.7239
SFA (g)	29.2 ± 6.8	30.4 (15.6–41.4)	29.6 ± 8.6	29.6 (11.1–53.0)	0.8622
SFA (% of energy value)	12.1 ± 2.5	13.2 * (4.7–15.3)	12.4 ± 2.5	12.2 (5.3–19.0)	0.7744
MUFA (g)	29.1 ± 10.5	26.1 * (18.4–60.2)	28.3 ± 9.7	26.8 (10.7–60.2)	0.9416
MUFA (% of energy value)	11.9 ± 3.5	11.0 * (8.4–24.7)	12.0 ± 3.5	12.2 (5.2–20.0)	0.7139
PUFA (g)	10.7 ± 5.8	8.4 * (6.1–27.7)	10.4 ± 5.6	9.0 * (4.4–27.3)	0.6941
PUFA (% of energy value)	4.3 ± 2.1	3.6 * (2.6–11.4)	4.5 ± 2.4	3.7 * (2.1–12.0)	0.8259
Protein (g)	88.6 ± 13.1	89.8 (63.7–111.2)	83.7 ± 13.1	85.0 (54.2–122.4)	0.1504
Protein (% of energy value)	16.2 ± 2.0	16.9 * (12.4–18.6)	15.9 ± 2.2	15.7 (11.8–22.3)	0.3753
Total carbohydrates (g)	300.1 ± 44.4	315.8 (181.7–359.2)	302.2 ± 60.8	296.3 (180.5–458.0)	0.8807
Carbohydrates (% of energy value)	53.1 ± 7.1	53.5 * (30.4–69.8)	52.7 ± 7.4	53.6 (38.0–67.5)	0.9097

* the distribution different than normal (verified using Shapiro-Wilk test—*p* ≤ 0.05); ** in the case of the normal distribution, a Student’s *t*-test was applied and in the case of a distribution different than the normal, the Mann–Whitney U test was applied; BM—body mass; SFA—saturated fatty acids; MUFA—monounsaturated fatty acids; PUFA—polyunsaturated fatty acids.

**Table 4:** Between-group comparisons of the percentage of individuals characterized by the recommended lipoprotein cholesterol fraction levels (chi^2^ test).

Blood Level *	Age < 40 Years		Age ≥ 40 Years		*p*
	Recommended Blood Level	Atherogenic Potential of Blood Level	Recommended Blood Level	Atherogenic Potential of Blood Level	
Total cholesterol (mg/dL)	6 (25.0%)	18 (75.0%)	11 (26.2%)	31 (73.8%)	1.0000
LDL cholesterol (mg/dL)	4 (16.7%)	20 (83.3%)	16 (38.1%)	26 (61.9%)	0.1226
HDL cholesterol (mg/dL)	20 (83.3%)	4 (16.7%)	36 (85.7%)	6 (14.3%)	1.0000
TC/HDL ratio (–)	11 (45.8%)	13 (54.2%)	28 (66.7%)	14 (33.3%)	0.1628
HDL/LDL ratio (–)	19 (79.2%)	5 (20.8%)	39 (92.9%)	3 (7.1%)	0.4084
Triglyceride (mg/dL)	16 (66.7%)	8 (33.3%)	29 (69.0%)	13 (30.9%)	1.0000
AIP (–)	4 (16.7%)	20 (83.3%)	7 (16.7%)	35 (83.3%)	1.0000

* the recommended blood level, applied as follows: Total cholesterol < 190 mg/dL [24]; LDL cholesterol < 115 mg/dL [24]; HDL cholesterol > 45 mg/dL [24]; TC/HDL ratio < 4.0 [25]; HDL/LDL ratio > 0.3 [26]; triglyceride < 150 mg/dL [24]; AIP <0.11 [27]; LDL—low-density lipoprotein; HDL—high-density lipoprotein; AIP—Atherogenic Index of Plasma.

**Table 5:** Analysis of the correlation between total cholesterol (TC) blood level and dietary fat intake in overweight women, stratified by age.

Dietary Intake	Age < 40 Years		Age ≥ 40 Years	
	*p*	*R*	*p*	*R*
Fat (g)	0.944 *	−0.0152	0.538 *	0.0979
Fat (% of energy value)	0.989 *	−0.0030	0.626 *	0.0775
SFA (g)	0.655	−0.0962	0.436	0.1234
SFA (% of energy value)	0.515 *	−0.1396	0.509 *	0.1047
MUFA (g)	0.715 *	0.0787	0.729 *	0.0552
MUFA (% of energy value)	0.963 *	−0.0100	0.790 *	0.0423
PUFA (g)	0.765 *	0.0643	0.209 *	0.1978
PUFA (% of energy value)	0.963 *	−0.0100	0.248 *	0.1822
Alpha-linolenic acid (ALA—18:3) **	0.773 *	0.0621	0.113 *	−0.3390
Eicosapentaenoic acid (EPA—20:5) **	0.590 *	0.1158	0.774 *	−0.0633
Docosahexaenoic (DHA—22:6) **	0.248 *	0.2453	0.868 *	0.0366
Total n-6 PUFA (g) ***	0.878 *	0.0331	0.778	0.0623
Cholesterol (mg)	0.323	−0.2105	0.746	0.0514
PUFA/SFA (–)	0.761 *	0.0657	0.640 *	0.0744
Trans fatty acids (g)	0.378	0.1882	0.878	0.0243

* in the case of the distribution different than normal (verified using Shapiro-Wilk test—*p* ≤ 0.05), the Spearman’s rank correlation was applied (for the normal distribution—the Pearson correlation was applied); ** n-3 PUFA: Alpha-linolenic acid (ALA—18:3), eicosapentaenoic acid (EPA—20:5), docosahexaenoic acid (DHA—22:6); *** total n-6 PUFA calculated as a sum of linoleic acid (LA—18:2), gamma-linolenic acid (GLA—18:3) and arachidonic acid (ARA—20:4); SFA—saturated fatty acids; MUFA—monounsaturated fatty acids; PUFA—polyunsaturated fatty acids.

**Table 6:** Analysis of the correlation between low-density lipoprotein (LDL) cholesterol blood level and dietary fat intake in overweight women, stratified by age.

Dietary Intake	Age < 40 Years		Age ≥ 40 Years	
	*p*	*R*	*p*	*R*
Fat (g)	0.939 *	−0.0165	0.594 *	0.0847
Fat (% of energy value)	0.527 *	−0.1356	0.835 *	0.0332
SFA (g)	0.648	−0.0982	0.271	0.1739
SFA (% of energy value)	0.933 *	−0.0182	0.769 *	0.0466
MUFA (g)	0.680 *	−0.0886	0.791 *	0.0421
MUFA (% of energy value)	0.377 *	−0.1887	0.975 *	0.0049
PUFA (g)	0.506 *	−0.1426	0.412 *	0.1301
PUFA (% of energy value)	0.412 *	−0.1756	0.552 *	0.0943
Alpha-linolenic acid (ALA—18:3) **	0.593 *	−0.1148	0.325 *	−0.2147
Eicosapentaenoic acid (EPA—20:5) **	0.763 *	0.0648	0.235 *	−0.2576
Docosahexaenoic (DHA—22:6) **	0.273 *	0.2330	0.346 *	−0.2058
Total n-6 PUFA (g) ***	0.384 *	−0.1861	0.762 *	−0.0668
Cholesterol (mg)	0.517	−0.1389	0.410	0.1305
PUFA/SFA (–)	0.530 *	−0.1348	0.939 *	−0.0121
Trans fatty acids (g)	0.297	0.2219	0.917	−0.0165

* in the case of the distribution different than normal (verified using Shapiro-Wilk test—*p* ≤ 0.05), the Spearman’s rank correlation was applied (for the normal distribution—the Pearson correlation was applied); ** n-3 PUFA: Alpha-linolenic acid (ALA—18:3), eicosapentaenoic acid (EPA—20:5), docosahexaenoic acid (DHA—22:6); *** total n-6 PUFA calculated as a sum of linoleic acid (LA—18:2), gamma-linolenic acid (GLA—18:3) and arachidonic acid (ARA—20:4); SFA—saturated fatty acids; MUFA—monounsaturated fatty acids; PUFA—polyunsaturated fatty acids.

**Table 7:** Analysis of the correlation between high-density lipoprotein (HDL) cholesterol blood level and dietary fat intake in overweight women, stratified by age.

Dietary Intake	Age < 40 Years		Age ≥ 40 Years	
	*p*	*R*	*p*	*R*
Fat (g)	0.560 *	0.1253	0.540 *	0.0972
Fat (% of energy value)	0.912 *	0.0239	0.626 *	0.0775
SFA (g)	0.230	−0.2544	0.179	−0.2116
SFA (% of energy value)	0.204 *	−0.2689	0.509 *	0.1047
MUFA (g)	0.240 *	0.2493	0.729 *	0.0552
MUFA (% of energy value)	0.722 *	0.0766	0.790 *	0.0424
PUFA (g)	0.060 *	0.3889	0.072 *	0.2807
PUFA (% of energy value)	0.214 *	0.2632	0.049 *	0.3056
Alpha-linolenic acid (ALA—18:3) **	0.293 *	0.2236	0.625 *	0.1077
Eicosapentaenoic acid (EPA—20:5) **	0.180 *	0.2831	0.273 *	0.2387
Docosahexaenoic (DHA—22:6) **	0.259 *	0.2397	0.353 *	0.2031
Total n-6 PUFA (g) ***	0.067 *	0.3802	0.530 *	0.1379
Cholesterol (mg)	0.672	−0.0910	0.773	0.0459
PUFA/SFA (–)	0.085 *	0.3589	0.640 *	0.0740
Trans fatty acids (g)	0.452	0.1610	0.831	0.0339

* in the case of the distribution different than normal (verified using Shapiro-Wilk test—*p* ≤ 0.05), the Spearman’s rank correlation was applied (for the normal distribution—the Pearson correlation was applied); ** n-3 PUFA: Alpha-linolenic acid (ALA—18:3), eicosapentaenoic acid (EPA—20:5), docosahexaenoic acid (DHA—22:6); *** total n-6 PUFA calculated as a sum of linoleic acid (LA—18:2), gamma-linolenic acid (GLA—18:3) and arachidonic acid (ARA—20:4); SFA—saturated fatty acids; MUFA—monounsaturated fatty acids; PUFA—polyunsaturated fatty acids.

**Table 8:** Analysis of the correlation between total cholesterol to high-density lipoprotein cholesterol blood ratio (TC/HDL) and dietary fat intake in overweight women, stratified by age.

Dietary Intake	Age < 40 Years		Age ≥ 40 Years	
	*p*	*R*	*p*	*R*
Fat (g)	0.765 *	–0.0640	0.577 *	–0.0886
Fat (% of energy value)	0.913 *	–0.0230	0.213 *	–0.196
SFA (g)	0.590	0.1156	0.147	0.2276
SFA (% of energy value)	0.525 *	0.1365	0.350 *	0.1480
MUFA (g)	0.565 *	–0.1235	0.189 *	–0.2065
MUFA (% of energy value)	0.885 *	–0.0313	0.072 *	–0.3806
PUFA (g)	0.159 *	0.2965	0.229 *	–0.1899
PUFA (% of energy value)	0.250 *	0.2443	0.130 *	–0.2372
Alpha-linolenic acid (ALA—18:3) **	0.384 *	–0.1861	0.188 *	–0.2846
Eicosapentaenoic acid (EPA—20:5) **	0.566 *	–0.1232	0.356 *	–0.2018
Docosahexaenoic (DHA—22:6) **	0.878 *	0.0330	0.553 *	–0.1304
Total n-6 PUFA (g) ***	0.175 *	–0.2861	0.541 *	–0.1344
Cholesterol (mg)	0.916	–0.0228	0.980	–0.0041
PUFA/SFA (–)	0.198 *	–0.2740	0.017 *	–0.3680
Trans fatty acids (g)	0.320	0.2120	0.633	0.0758

* in the case of the distribution different than normal (verified using Shapiro-Wilk test—*p* ≤ 0.05), the Spearman’s rank correlation was applied (for the normal distribution—the Pearson correlation was applied); ** n-3 PUFA: Alpha-linolenic acid (ALA—18:3), eicosapentaenoic acid (EPA—20:5), docosahexaenoic acid (DHA—22:6); *** total n-6 PUFA calculated as a sum of linoleic acid (LA—18:2), gamma-linolenic acid (GLA—18:3) and arachidonic acid (ARA—20:4); SFA—saturated fatty acids; MUFA—monounsaturated fatty acids; PUFA—polyunsaturated fatty acids.

**Table 9:** Analysis of the correlation between high-density lipoprotein cholesterol to low-density lipoprotein cholesterol blood ratio (HDL/LDL) and dietary fat intake in overweight women, stratified by age.

Dietary Intake	Age < 40 Years		Age ≥ 40 Years	
	*p*	*R*	*p*	*R*
Fat (g)	0.692 *	0.0852	0.897 *	0.0206
Fat (% of energy value)	0.680 *	0.0887	0.394 *	0.1349
SFA (g)	0.778 *	0.0609	0.270 *	−0.1743
SFA (% of energy value)	0.725 *	−0.0757	0.437 *	−0.1232
MUFA (g)	0.426 *	0.1704	0.448 *	0.1201
MUFA (% of energy value)	0.599 *	0.1130	0.195 *	0.2041
PUFA (g)	0.159 *	0.2965	0.429 *	0.1253
PUFA (% of energy value)	0.250 *	0.2443	0.254 *	0.1799
Alpha-linolenic acid (ALA—18:3) **	0.332 *	0.2070	0.376 *	0.1937
Eicosapentaenoic acid (EPA—20:5) **	0.458 *	0.1589	0.300 *	0.2261
Docosahexaenoic (DHA—22:6) **	0.997 *	0.0009	0.371 *	0.1956
Total n-6 PUFA (g) ***	0.146 *	0.3061	0.347 *	0.2055
Cholesterol (mg)	0.710 *	0.0800	0.855 *	−0.0290
PUFA/SFA (–)	0.198 *	0.2721	0.049 *	0.3062
Trans fatty acids (g)	0.181 *	0.2826	0.994 *	−0.0012

* in the case of the distribution different than normal (verified using Shapiro-Wilk test—*p* ≤ 0.05), the Spearman’s rank correlation was applied (for the normal distribution—the Pearson correlation was applied); ** n-3 PUFA: Alpha-linolenic acid (ALA—18:3), eicosapentaenoic acid (EPA—20:5), docosahexaenoic acid (DHA—22:6); *** total n-6 PUFA calculated as a sum of linoleic acid (LA—18:2), gamma-linolenic acid (GLA—18:3) and arachidonic acid (ARA—20:4); SFA—saturated fatty acids; MUFA—monounsaturated fatty acids; PUFA—polyunsaturated fatty acids.

**Table 10:** Analysis of the correlation between triglyceride blood level and dietary fat intake in overweight women, stratified by age.

Dietary Intake	Age < 40 Years		Age ≥ 40 Years	
	*p*	*R*	*p*	*R*
Fat (g)	0.085 *	−0.3583	0.077 *	−0.2756
Fat (% of energy value)	0.682 *	0.0882	0.322 *	−0.1565
SFA (g)	0.440 *	0.1652	0.240 *	−0.1854
SFA (% of energy value)	0.078 *	0.3666	0.410 *	0.1306
MUFA (g)	0.085 *	0.3592	0.643 *	0.0737
MUFA (% of energy value)	0.659 *	−0.0948	0.141 *	−0.2311
PUFA (g)	0.915 *	0.0230	0.082 *	−0.2717
PUFA (% of energy value)	0.310 *	−0.2161	0.281 *	−0.1704
Alpha-linolenic acid (ALA—18:3) **	0.441 *	−0.1648	0.700 *	−0.0850
Eicosapentaenoic acid (EPA—20:5) **	0.456 *	−0.1598	0.093 *	−0.3583
Docosahexaenoic (DHA—22:6) **	0.402 *	−0.1792	0.258 *	−0.2457
Total n-6 PUFA (g) ***	0.388 *	−0.1844	0.884 *	−0.0321
Cholesterol (mg)	0.228 *	−0.2557	0.206 *	−0.1990
PUFA/SFA (–)	0.697 *	−0.0831	0.771 *	−0.0463
Trans fatty acids (g)	0.191 *	0.2766	0.994 *	0.0012

* in the case of the distribution different than normal (verified using Shapiro-Wilk test—*p* ≤ 0.05), the Spearman’s rank correlation was applied (for the normal distribution—the Pearson correlation was applied); ** n-3 PUFA: Alpha-linolenic acid (ALA—18:3), eicosapentaenoic acid (EPA—20:5), docosahexaenoic acid (DHA—22:6); *** total n-6 PUFA calculated as a sum of linoleic acid (LA—18:2), gamma-linolenic acid (GLA—18:3) and arachidonic acid (ARA—20:4); SFA—saturated fatty acids; MUFA—monounsaturated fatty acids; PUFA—polyunsaturated fatty acids.

**Table 11:** Analysis of the correlation between the Atherogenic Index of Plasma (AIP) and dietary fat intake in overweight women, stratified by age.

Dietary Intake	Age < 40 Years		Age ≥ 40 Years	
	*p*	*R*	*p*	*R*
Fat (g)	0.567 *	0.0957	0.180 *	−0.2109
Fat (% of energy value)	0.565 *	0.1235	0.133 *	−0.2357
SFA (g)	0.040	0.4223	0.315	0.1589
SFA (% of energy value)	0.031 *	0.4417	0.493 *	0.1088
MUFA (g)	0.571 *	−0.1217	0.079 *	−0.2738
MUFA (% of energy value)	0.984 *	−0.0043	0.384 *	−0.3206
PUFA (g)	0.107 *	−0.3373	0.100 *	−0.2569
PUFA (% of energy value)	0.094 *	−0.3486	0.067 *	−0.2856
Alpha-linolenic acid (ALA—18:3) **	0.275 *	−0.2322	0.409 *	−0.1808
Eicosapentaenoic acid (EPA—20:5) **	0.316 *	−0.2137	0.109 *	−0.3428
Docosahexaenoic (DHA—22:6) **	0.338 *	−0.2043	0.224 *	−0.2638
Total n-6 PUFA (g) ***	0.145 *	−0.3070	0.680 *	−0.0909
Cholesterol (mg)	0.636	−0.1019	0.493	−0.1087
PUFA/SFA (–)	0.019 *	−0.4757	0.011 *	−0.3901
Trans fatty acids (g)	0.282	0.2287	0.746	0.0515

* in the case of the distribution different than normal (verified using Shapiro-Wilk test—*p* ≤ 0.05), the Spearman’s rank correlation was applied (for the normal distribution—the Pearson correlation was applied); ** n-3 PUFA: Alpha-linolenic acid (ALA—18:3), eicosapentaenoic acid (EPA—20:5), docosahexaenoic acid (DHA—22:6); *** total n-6 PUFA calculated as a sum of linoleic acid (LA—18:2), gamma-linolenic acid (GLA—18:3) and arachidonic acid (ARA—20:4); SFA—saturated fatty acids; MUFA—monounsaturated fatty acids; PUFA—polyunsaturated fatty acids.

## Supplementary Materials

The following are available online at http://www.mdpi.com/1660-4601/15/11/2530/s1, Table S1: Between-group comparison of the food products intake in a diet of participants, Table S2: Analysis of the correlation between total cholesterol blood level and food products intake in overweight women, stratified by age, Table S3: Analysis of the correlation between low-density lipoprotein (LDL) cholesterol blood level and food products intake in overweight women, stratified by age, Table S4: Analysis of the correlation between high-density lipoprotein (HDL) cholesterol blood level and food products intake in overweight women, stratified by age, Table S5: Analysis of the correlation between total cholesterol to high-density lipoprotein cholesterol blood ratio (TC/HDL) and food products intake in overweight women, stratified by age, Table S6: Analysis of the correlation between high-density lipoprotein cholesterol to low-density lipoprotein cholesterol blood ratio (HDL/LDL) and food products intake in overweight women, stratified by age, Table S7: Analysis of the correlation between triglyceride blood level and food products intake in overweight women, stratified by age, Table S8: Analysis of the correlation between the Atherogenic Index of Plasma (AIP) and food products intake in overweight women, stratified by age.

## References

[1] Yngve A. (2009). A Historical perspective of the understanding of the link between diet and coronary heart disease. Am. J. Lifestyle Med..

[2] The American Heart Association’s Diet and Lifestyle Recommendations. http://www.heart.org/HEARTORG/HealthyLiving/Diet-and-Lifestyle-Recommendations_UCM_305855_Article.jsp#.W2WF9jkyUkl.

[3] Siri-Tarino P.W., Sun Q., Hu F.B., Krauss R.M. (2010). Meta-analysis of prospective cohort studies evaluating the association of saturated fat with cardiovascular disease. Am. J. Clin. Nutr..

[4] Clifton P.M., Keogh J.B. (2017). A systematic review of the effect of dietary saturated and polyunsaturated fat on heart disease. Nutr. Metab. Cardiovasc. Dis..

[5] De Souza R.J., Mente A., Maroleanu A., Cozma A.I., Ha V., Kishibe T., Uleryk E., Budylowski P., Schünemann H., Beyene J. (2015). Intake of saturated and trans unsaturated fatty acids and risk of all cause mortality, cardiovascular disease, and type 2 diabetes: Systematic review and meta-analysis of observational studies. BMJ.

[6] Clarke R., Frost C., Collins R., Appleby P., Peto R. (1997). Dietary lipids and blood cholesterol: Quantitative meta-analysis of metabolic ward studies. BMJ.

[7] Hannon B.A., Thompson S.V., An R., Teran-Garcia M. (2017). Clinical outcomes of dietary replacement of saturated fatty acids with unsaturated fat sources in adults with overweight and obesity: A systematic review and meta-analysis of randomized control trials. Ann. Nutr. Metab..

[8] Burke G.L., Bertoni A.G., Shea S., Tracy R., Watson K.E., Blumenthal R.S., Chung H., Carnethon M.R. (2008). The impact of obesity on cardiovascular disease risk factors and subclinical vascular disease: The multi-ethnic study of atherosclerosis. Arch. Intern. Med..

[9] Khan S.S., Ning H., Wilkins J.T., Allen N., Carnethon M., Berry J.D., Sweis R.N., Lloyd-Jones D.M. (2018). Association of Body Mass Index with lifetime risk of cardiovascular disease and compression of morbidity. JAMA Cardiol..

[10] (2016). WHO, International Statistical Classification of Diseases and Related Health Problems 10th Revision, ICD-10 Version. http://apps.who.int/classifications/icd10/browse/2016/en.

[11] Frankenfield D.C., Muth E.R., Rowe W.A. (1998). The Harris-Benedict studies of human basal metabolism: History and limitations. J. Am. Diet. Assoc..

[12] Diederichs C., Neuhauser H., Rücker V., Busch M.A., Keil U., Fitzgerald A.P., Heuschmann P.U. (2018). Predicted 10-year risk of cardiovascular mortality in the 40 to 69 year old general population without cardiovascular diseases in Germany. PLoS ONE.

[13] Lind L., Sundström J., Ärnlöv J., Lampa E. (2018). Impact of aging on the strength of cardiovascular risk factors: A longitudinal study over 40 years. J. Am. Heart Assoc..

[14] Lloyd-Jones D.M., Larson M.G., Beiser A., Levy D. (1999). Lifetime risk of developing coronary heart disease. Lancet.

[15] Wilson P.W.F. Overview of Established Risk Factors for Cardiovascular Disease. https://www.uptodate.com/contents/cardiovascular-disease-risk-assessment-for-primary-prevention-risk-calculators.

[16] ISAK (2001). International Standards for Anthropometric Assessment. Potchefstroom, South Africa: International Society for the Advancement of Kinanthropometry. http://www.ceap.br/material/MAT17032011184632.pdf.

[17] Branca F., Nikogosian H., Lobstein T., World Health Organization (2007). The Challenge of Obesity in the WHO European Region and the Strategies for Response.

[18] Szponar L., Wolnicka K., Rychlik E. (2000). Atlas of Food Products and Dishes Portion Sizes.

[19] Wajszczyk B., Chwojnowska Z., Nasiadko D., Rybaczuk M. (2015). Dieta 5.0 Software for Individual and Group Nutrition Assessment and Diet Planning.

[20] Kunachowicz H., Nadolna I., Przygoda B., Iwanow K. (2017). Food Composition Tables.

[21] United States Department of Agriculture Agricultural Research Service USDA Food Composition Databases. https://ndb.nal.usda.gov/ndb/nutrients/report/nutrientsfrm?max=25&offset=0&totCount=0&nutrient1=605&nutrient2=&subset=0&sort=c&measureby=g].

[22] Friedewald W.T., Levy R.I., Fredrickson D.S. (1972). Estimation of the concentration of low-density lipoprotein cholesterol in plasma without use of the preparative ultracentrifuge. Clin. Chem..

[23] Dobiasova M. (2006). AIP—Atherogenic index of plasma as a significant predictor of cardiovascular risk: From research to practice. Vnitr. Lek..

[24] Perk J., De Backer G., Gohlke H., Graham I., Reiner Z., Verschuren M., Albus C., Benlian P., Boysen G., Cifkova R. (2012). European Guidelines on cardiovascular disease prevention in clinical practice (version 2012). The Fifth Joint Task Force of the European Society of Cardiology and Other Societies on Cardiovascular Disease Prevention in Clinical Practice (Constituted by Representatives of Nine Societies and by Invited Experts). Eur. Heart J..

[25] Criqui M.H., Golomb B.A. (1998). Epidemiologic aspects of lipid abnormalities. Am. J. Med..

[26] Corrigan M.L., Escuro A.A., Kirby D.F. (2013). Obesity and Stroke. Handbook of Clinical Nutrition and Stroke.

[27] Rosolova H., Dobiasova M., Soska V., Blaha V., Ceska R., Nussbaumerova B., Pelikanova T., Soucek M. (2014). Combined therapy of mixed dyslipidemia in patients with high cardiovascular risk and changes in the lipid target values and atherogenic index of plasma. Cor et Vasa.

[28] Paige E., Barrett J., Pennells L., Sweeting M., Willeit P., Di Angelantonio E., Gudnason V., Nordestgaard B.G., Psaty B.M., Goldbourt U. (2017). Use of repeated blood pressure and cholesterol measurements to improve cardiovascular disease risk prediction: An individual-participant-data meta-analysis. Am. J. Epidemiol..

[29] Robinson J.G., Wang S., Smith B.J., Jacobson T.A. (2009). Meta-analysis of the relationship between non-high-density lipoprotein cholesterol reduction and coronary heart disease risk. J. Am. Coll. Cardiol..

[30] Peters S.A., Singhateh Y., Mackay D., Huxley R.R., Woodward M. (2016). Total cholesterol as a risk factor for coronary heart disease and stroke in women compared with men: A systematic review and meta-analysis. Atherosclerosis.

[31] Di Angelantonio E., Bhupathiraju S.N., Wormser D., Gao P., Kaptoge S., Berrington de Gonzalez A., Cairns B.J., Huxley R., Jackson C.L., Global BMI Mortality Collaboration (2016). Body-mass index and all-cause mortality: Individual-participant-data meta-analysis of 239 prospective studies in four continents. Lancet.

[32] Kong K.A., Park J., Hong S., Hong Y.S., Sung Y.-A., Lee H. (2017). Associations between body mass index and mortality or cardiovascular events in a general Korean population. PLoS ONE.

[33] Cho Y.G. (2017). Cardiovascular risk assessment based on combined Body Mass Index and Waist Circumference in Korean adults. Korean J. Fam. Med..

[34] Aune D., Sen A., Norat T., Janszky I., Romundstad P., Tonstad S., Vatten L.J. (2016). Body Mass Index, abdominal fatness, and heart failure incidence and mortality: A systematic review and dose-response meta-analysis of prospective studies. Circulation.

[35] Burillo E., Martín-Fuentes P., Mateo-Gallego R., Baila-Rueda L., Cenarro A., Ros E., Civeira F. (2012). Omega-3 fatty acids and HDL. How do they work in the prevention of cardiovascular disease?. Curr. Vasc. Pharmacol..

[36] Zuliani G., Galvani M., Leitersdorf E., Volpato S., Cavalieri M., Fellin R. (2009). The role of polyunsaturated fatty acids (PUFA) in the treatment of dyslipidemias. Curr. Pharm. Des..

[37] Sanders T.A., Hochland M.C. (1983). A comparison of the influence on plasma lipids and platelet function of supplements of omega 3 and omega 6 polyunsaturated fatty acids. Br. J. Nutr..

[38] Simons L.A., Hickie J.B., Balasubramaniam S. (1985). On the effects of dietary n-3 fatty acids (Maxepa) on plasma lipids and lipoproteins in patients with hyperlipidaemia. Atherosclerosis.

[39] Pizzini A., Lunger L., Demetz E., Hilbe R., Weiss G., Ebenbichler C., Tancevski I. (2017). The role of omega-3 fatty acids in reverse cholesterol transport: A review. Nutrients.

[40] Mensink R.P., Zock P.L., Kester A.D., Katan M.B. (2003). Effects of dietary fatty acids and carbohydrates on the ratio of serum total to HDL cholesterol and on serum lipids and apolipoproteins: A meta-analysis of 60 controlled trials. Am. J. Clin. Nutr..

[41] Bradberry J.C., Hilleman D.E. (2013). Overview of omega-3 fatty acid therapies. Pharm. Ther..

[42] Ooi E.M., Watts G.F., Ng T.W., Barrett P.H. (2015). Effect of dietary fatty acids on human lipoprotein metabolism: A comprehensive update. Nutrients.

[43] Grimsgaard S., Bonaa K.H., Hansen J.B., Nordøy A. (1997). Highly purified eicosapentaenoic acid and docosahexaenoic acid in humans have similar triacylglycerol-lowering effects but divergent effects on serum fatty acids. Am. J. Clin. Nutr..

[44] Egert S., Kannenberg F., Somoza V., Erbersdobler H.F., Wahrburg U. (2009). Dietary alpha-linolenic acid, EPA, and DHA have differential effects on LDL fatty acid composition but similar effects on serum lipid profiles in normolipidemic humans. J. Nutr..

[45] Cottin S.C., Sanders T.A., Hall W.L. (2011). The differential effects of EPA and DHA on cardiovascular risk factors. Proc. Nutr. Soc..

[46] Harris W.S., Pottala J.V., Varvel S.A., Borowski J.J., Ward J.N., McConnell J.P. (2013). Erythrocyte omega-3 fatty acids increase and linoleic acid decreases with age: Observations from 160,000 patients. Prostaglandins Leukot. Essent. Fat. Acids.

[47] Farvid M.S., Ding M., Pan A., Sun Q., Chiuve S.E., Steffen L.M., Willett W.C., Hu F.B. (2014). Dietary linoleic acid and risk of coronary heart disease: A systematic review and meta-analysis of prospective cohort studies. Circulation.

[48] Cai G., Xue S., Lu W. (2017). The Atherogenic Index of plasma is a strong and independent predictor for coronary artery disease in the Chinese Han population. Medicine.

[49] Niroumand S., Khajedaluee M., Khadem-Rezaiyan M., Abrishami M., Juya M., Khodaee G., Dadgarmoghaddam M. (2015). Atherogenic Index of Plasma (AIP): A marker of cardiovascular disease. Med. J. Islamic Repub. Iran.

[50] Gao M., Zheng Y., Zhang W., Cheng Y., Wang L., Qin L. (2017). Non-high-density lipoprotein cholesterol predicts nonfatal recurrent myocardial infarction in patients with ST segment elevation myocardial infarction. Lipids Health Dis..

[51] Zhu L., Lu Z., Zhu L., Ouyang X., Yang Y., He W., Feng Y., Yi F., Song Y. (2015). Lipoprotein ratios are better than conventional lipid parameters in predicting coronary heart disease in Chinese Han people. Kardiol. Pol..

[52] Dobiasova M., Frohlich J. (2001). The plasma parameter log (TG/HDL-C) as an atherogenic index: Correlation with lipoprotein particle size and esterification rate in apoB-lipoprotein-depleted plasma (FER(HDL)). Clin. Biochem..

[53] Shen S., Lu Y., Qi H., Li F., Shen Z., Wu L., Yang C., Wang L., Shui K., Wang Y. (2016). Association between ideal cardiovascular health and the atherogenic index of plasma. Medicine.

[54] Angus J.E., Rukholm E., Michel I., Larocque S., Seto L., Lapum J., Timmermans K., Chevrier-Lamoureux R., Nolan R.P. (2009). Context and cardiovascular risk modification in two regions of Ontario, Canada: A photo elicitation study. Int. J. Environ. Res. Public Health.

[55] Heitmann B.L., Lissner L. (1995). Dietary underreporting by obese individuals—Is it specific or non-specific?. Br. Med. J..

