# Supplementary material for: Intake of Saturated Fatty Acids Affects Atherogenic Blood Properties in Young, Caucasian, Overweight Women Even without Influencing Blood Cholesterol
Source: Int J Environ Res Public Health. 2018 Nov 12;15(11):2530. doi: 10.3390/ijerph15112530 (PMC6267335; doi:10.3390/ijerph15112530)
Supplement: Supplementary file 1 [file ijerph-15-02530-s001.pdf]

Intake of saturated fatty acids affects atherogenic blood properties in young, Caucasian, overweight women even without influencing blood cholesterol

Jadwiga Hamułka [1]*, Dominika Głąbska [2], Dominika Guzek [3], Agnieszka Białkowska [1,4] and Agnieszka Sulich [1]

Table S1. Between-group comparison of the food products intake in a diet of participants.

Food Products	Age < 40 Years		Age ≥ 40 Years		p **
	Mean ± SD	Median (range)	Mean ± SD	Median (range)	
Dairy beverages (g)	230.0 ± 164.1	189.2 (6.7-573.3)	210.4 ± 152.6	198.3 (0.0-623.3)	0.6261
Low-fat dairy products (g)	47.8 ± 44.5	34.4 * (0.0-123.3)	63.5 ± 53.7	50.0 * (0.0-183.3)	0.2381
High-fat dairy products (g)	21.8 ± 17.1	18.7 * (0.0-67.0)	21.6 ± 21.3	15.8 * (0.0-90.0)	0.7189
Meat (g)	90.4 ± 49.4	84.1 (0.0-240.0)	81.8 ± 38.3	76.7 (0.0-183.8)	0.4353
Meat products (g)	66.2 ± 33.3	63.3 (13.3-160.8)	52.9 ± 32.9	57.5 (0.0-135.0)	0.1188
Fish and fish products (g)	11.8 ± 23.4	0.0 * (0.0-90.0)	10.6 ± 19.9	0.0 * (0.0-70.0)	0.9522
Egg (g)	27.0 ± 17.6	25.2 (0.0-55.3)	25.7 ± 19.6	18.8 * (0.0-82.5)	0.5266
Plant fats (g)	19.7 ± 14.3	20.3 (0.0-55.0)	19.7 ± 17.1	16.3 * (0.0-72.3)	0.6941
Dairy fats (g)	28.6 ± 19.3	27.8 (0.0-70.0)	30.1 ± 19.7	27.1 * (2.5-113.8)	0.8311

* the distribution different than normal (verified using Shapiro-Wilk test – $p \leq 0.05$); ** in the case of the normal distribution Student's t-test was applied and distribution different than normal, the Mann–Whitney U test was applied.

Table S2. Analysis of the correlation between total cholesterol (TC) blood level and food products intake in overweight women, stratified by age.

Food Products	Age < 40 Years		Age ≥ 40 Years	
	p	R	p	R
Dairy beverages	0.231	0.2539	0.032	–0.3315
Low–fat dairy products	0.870 *	–0.0353	0.492 *	0.1090
High–fat dairy products	0.216 *	–0.2621	0.381 *	0.1386
Meat	0.197	–0.2728	0.009	0.4009
Meat products	0.012	0.5065	0.349	–0.1481
Fish and fish products	0.839 *	0.0439	0.370 *	0.1418
Egg	0.743	–0.0707	0.801 *	–0.0401
Plant fats	0.321	–0.2117	0.093 *	–0.2628
Dairy fats	0.097	0.3470	0.465 *	0.1157

* in the case of the distribution different than normal (verified using Shapiro-Wilk test – $p \leq 0.05$), the Spearman's rank correlation was applied (for the normal distribution – the Pearson correlation was applied).

Table S3. Analysis of the correlation between low-density lipoprotein (LDL) cholesterol blood level and food products intake in overweight women, stratified by age.

Food Products	Age < 40 Years		Age ≥ 40 Years	
	p	R	*p*	R
Dairy beverages	0.167	0.2916	0.025	–0.3445
Low–fat dairy products	0.884 *	–0.0314	0.528*	0.1000
High–fat dairy products	0.214 *	–0.2631	0.405*	0.1318
Meat	0.159	–0.2966	0.005	0.4250
Meat products	0.028	0.4481	0.201	–0.2014
Fish and fish products	0.839 *	0.0439	0.418 *	0.1284
Egg	0.761	–0.0654	0.764 *	–0.0478
Plant fats	0.464	–0.1568	0.084 *	–0.2698
Dairy fats	0.087	0.3569	0.468 *	0.1150

* in the case of the distribution different than normal (verified using Shapiro-Wilk test – $p \leq 0.05$), the Spearman's rank correlation was applied (for the normal distribution – the Pearson correlation was applied).

Table S4. Analysis of the correlation between high-density lipoprotein (HDL) cholesterol blood level and food products intake in overweight women, stratified by age.

Food Products	Age < 40 Years		Age ≥ 40 Years	
	p	R	*p*	R
Dairy beverages	0.393	0.1826	0.025	–0.3445
Low–fat dairy products	0.944 *	–0.0150	0.501 *	0.1068
High–fat dairy products	0.220 *	–0.2598	0.399 *	0.1336
Meat	0.186	–0.2796	0.009	0.3981
Meat products	0.057	0.3932	0.418	–0.1284
Fish and fish products	0.839 *	0.0458	0.409 *	0.1309
Egg	0.825	–0.0476	0.772 *	–0.0461
Plant fats	0.544	–0.1304	0.096 *	–0.2604
Dairy fats	0.115	0.3299	0.455 *	0.1184

* in the case of the distribution different than normal (verified using Shapiro-Wilk test – $p \leq 0.05$), the Spearman's rank correlation was applied (for the normal distribution – the Pearson correlation was applied).

Table S5. Analysis of the correlation between total cholesterol to high-density lipoprotein cholesterol blood ratio (TC/HDL) and food products intake in overweight women, stratified by age.

Food Products	Age < 40 Years		Age ≥ 40 Years	
	p	R	*p*	R
Dairy beverages	0.425	0.1707	0.068	–0.2848
Low–fat dairy products	0.884 *	–0.0314	0.500 *	0.1071
High–fat dairy products	0.214 *	–0.2631	0.397 *	0.1341
Meat	0.314	–0.2147	0.009	0.3964
Meat products	0.040	0.4229	0.273	–0.1732
Fish and fish products	0.839 *	0.0439	0.402 *	0.1328
Egg	0.839	–0.0437	0.775 *	–0.0455
Plant fats	0.438	–0.1660	0.092 *	–0.2634
Dairy fats	0.154	0.3003	0.452 *	0.1184

* in the case of the distribution different than normal (verified using Shapiro-Wilk test – $p \leq 0.05$), the Spearman's rank correlation was applied (for the normal distribution – the Pearson correlation was applied).

Table S6. Analysis of the correlation between high-density lipoprotein cholesterol to low-density lipoprotein cholesterol blood ratio (HDL/LDL) and food products intake in overweight women, stratified by age.

**Food Products**	**Age < 40 Years**		**Age ≥ 40 Years**	
	p	**R**	***p***	**R**
Dairy beverages	0.407 *	0.1774	0.060 *	–0.2923
Low–fat dairy products	0.883 *	–0.0314	0.500 *	0.1071
High–fat dairy products	0.214 *	–0.2631	0.397 *	0.1341
Meat	0.284 *	–0.2279	0.007 *	0.4093
Meat products	0.207 *	0.2671	0.460 *	–0.1171
Fish and fish products	0.839 *	0.0439	0.402 *	0.1328
Egg	0.643 *	–0.0996	0.775*	–0.0455
Plant fats	0.386 *	–0.1854	0.092 *	–0.2634
Dairy fats	0.123 *	0.3235	0.452 *	0.1193

* in the case of the distribution different than normal (verified using Shapiro-Wilk test – $p \leq 0.05$), the Spearman's rank correlation was applied (for the normal distribution – the Pearson correlation was applied).

Table S7. Analysis of the correlation between triglyceride blood level and food products intake in overweight women, stratified by age.

**Food Products**	**Age < 40 Years**		**Age ≥ 40 Years**	
	p	**R**	***p***	**R**
Dairy beverages	0.382 *	0.1870	0.060 *	–0.2924
Low–fat dairy products	0.876 *	–0.0336	0.505 *	0.1057
High–fat dairy products	0.213 *	–0.2638	0.388 *	0.1366
Meat	0.301 *	–0.2200	0.007 *	0.4107
Meat products	0.219 *	0.2607	0.483 *	–0.1113
Fish and fish products	0.846 *	0.0420	0.397 *	0.1342
Egg	0.638 *	–0.1012	0.794 *	–0.0416
Plant fats	0.365 *	–0.1933	0.095 *	–0.0261
Dairy fats	0.117 *	0.3288	0.435 *	0.1237

* in the case of the distribution different than normal (verified using Shapiro-Wilk test – $p \leq 0.05$), the Spearman's rank correlation was applied (for the normal distribution – the Pearson correlation was applied).

Table S8. Analysis of the correlation between the Atherogenic Index of Plasma (AIP) and food products intake in overweight women, stratified by age.

**Food Products**	**Age < 40 Years**		**Age ≥ 40 Years**	
	p	**R**	***p***	**R**
Dairy beverages	0.238	0.2503	0.065	–0.2876
Low–fat dairy products	0.884 *	–0.0317	0.060 *	–0.2924
High–fat dairy products	0.213 *	–0.2683	0.500 *	0.1071
Meat	0.193	–0.2754	0.007	0.4105
Meat products	0.021	0.4691	0.301	–0.1634
Fish and fish products	0.846 *	0.0420	0.402 *	0.1328
Egg	0.476	–0.1526	0.775 *	–0.0455
Plant fats	0.375	–0.1895	0.092 *	–0.2634
Dairy fats	0.138	0.3119	0.452 *	0.1193

* in the case of the distribution different than normal (verified using Shapiro-Wilk test – $p \leq 0.05$), the Spearman's rank correlation was applied (for the normal distribution – the Pearson correlation was applied).
